# The bittersweet link between glucose metabolism, cellular microenvironment and viral infection

**DOI:** 10.1080/21505594.2025.2554302

**Published:** 2025-09-03

**Authors:** Xueao Liu, Li Chen, Honglin Niu, Yuhan Chen, Peiyao Chen, Lei Liu, Rui Wu

**Affiliations:** a Jiamusi University of Basic Medicine, Jiamusi University, Jiamusi, Heilongjiang, China; b The Affiliated First Hospital, Jiamusi University, Jiamusi, Heilongjiang, China

**Keywords:** Glucose metabolism, cellular microenvironment, viral infection, metabolic reprogramming, immune response

## Abstract

Viral particles and proteins released during infection profoundly reshape the cellular microenvironment by disrupting host signaling, triggering inflammation, and modulating immune responses. Glucose metabolism, a critical hub for energy production and biosynthesis, is highly susceptible to viral reprogramming. This review summarizes recent findings showing that diverse viruses, including influenza virus, Severe acute respiratory syndrome coronavirus 2 (SARS-CoV-2), and enteroviruses, manipulate glucose metabolic pathways to promote replication and evade immune surveillance. Specifically, viruses modulate glycolytic flux, alter the activity of key metabolic enzymes such as hexokinase (HK) and pyruvate kinase, and interfere with signaling networks like PI3K/Akt/mTOR and AMPK. These metabolic alterations further impact the immune landscape by regulating cytokine production, immune cell activation, and antiviral responses. Our analysis highlights a bidirectional interaction: while viruses hijack host glucose metabolism to favor their survival, metabolic changes also generate host-derived antiviral responses. This review highlights the bidirectional crosstalk between metabolic remodeling and microenvironmental changes during viral infection, underscoring the potential of metabolism-based antiviral strategies. A deeper understanding of these mechanisms may inform the development of more effective and targeted interventions against viral diseases.

## Introduction

In recent years, the role of glucose metabolism and the cellular microenvironment in viral infections has attracted widespread attention. Glucose metabolism involves three primary metabolic pathways: glycolysis, the pentose phosphate pathway (PPP), and the tricarboxylic acid (TCA) cycle. Studies have demonstrated that viral infections can reprogram host cell glucose metabolism to meet their replication and growth needs. For instance, hepatitis B virus (HBV), dengue virus (DENV), Epstein-Barr virus (EBV), and human immunodeficiency virus (HIV) can manipulate host cell glucose metabolism [1–4]. The cellular microenvironment includes cells and extracellular components that interact dynamically. The cellular microenvironment consists of various cell types, the extracellular matrix (ECM), extracellular regulatory molecules, and interstitial fluids. These components provide essential support, protection, connectivity, and nourishment to cells. Moreover, they are intricately linked to fundamental cellular activities, including proliferation, differentiation, adhesion, migration, and metabolism. Viral infections profoundly influence cellular metabolism, immune responses, and signaling pathways, leading to alterations in the cellular microenvironment.

Viral invasion activates immune responses, increasing immune cell recruitment and cytokine secretion. These immune cells rely heavily on glucose metabolism to maintain their survival and rapid proliferation [5–7]. Additionally, viruses can induce hypoxia through direct or indirect mechanisms. Under hypoxic conditions, cells upregulate glucose transporters (GLUTs) via hypoxia-inducible factor 1 (HIF-1), enhancing glucose uptake and shifting metabolism toward anaerobic glycolysis, which elevates intracellular lactate levels. This review focuses on the reciprocal regulation between glucose metabolism and the cellular microenvironment in the context of viral infection. Understanding this interplay is crucial for elucidating viral pathogenesis and identifying potential metabolic and microenvironmental targets for antiviral therapy.

## Cellular microenvironment and viral infection

### The regulatory mechanism of viruses on the cellular microenvironment

The ECM, a key structural component, comprises a complex network of proteins such as collagen, proteoglycans, elastin, and laminin (LN). Alterations in ECM composition can change tissue stiffness, affect cell adhesion, and disrupt intercellular communication. Extracellular regulatory molecules, including signaling molecules and cytokines, also play pivotal roles in microenvironmental regulation. Signaling molecules bind to specific receptors to transmit inter- and intracellular information. Cytokines, a subclass of signaling molecules, are secreted by immune cells (e.g. monocytes, macrophages, T cells, B cells, and natural killer cells) and nonimmune cells (e.g. endothelial cells, keratinocytes, and fibroblasts). These low-molecular-weight proteins regulate immune responses, promote angiogenesis, and mediate tissue repair.

Influenza A virus (IAV), hepatitis A virus (HAV), and HBV stimulate CD8^+^ T cell activation and differentiation into CTLs, which secrete cytokines that remodel the surrounding microenvironment [8–10]. SARS-CoV-2 engages with APCs, triggering antigen presentation to T and B lymphocytes. This interaction promotes T cell subtype differentiation and drives B cells to mature into plasma cells for antibody production. In severe cases, excessive immune responses contribute to lung tissue damage [11]. Infection with enterovirus 71 (EV71) alters ECM structure and extracellular regulatory factors, enhancing viral replication [12]. EBV induces dendritic cells to release type I IFNs, activating NK cells. It also stimulates monocytes and macrophages, reshaping the immune cell landscape [13].

**Figure 1.**
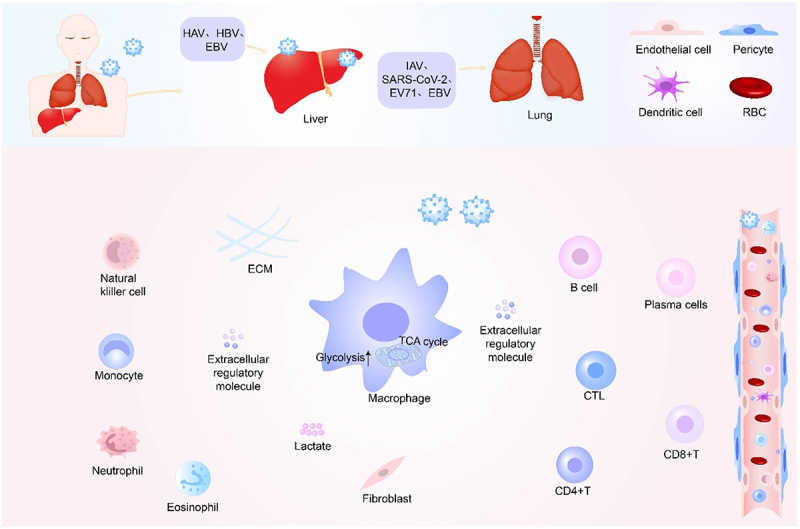
The impact of viruses on the cellular microenvironment.

ECM and extracellular signaling molecules are essential for maintaining the structural and functional integrity of the cellular microenvironment. During viral infection, both structural components and immune mediators are actively reprogrammed to facilitate viral survival. Viruses not only reshape the ECM and alter signaling networks but also modulate immune cell activation and cytokine secretion, ultimately creating a microenvironment conducive to viral replication and, in some cases, immune-mediated tissue damage (Figure 1).

When a virus infects the body, the cellular microenvironment changes, and in severe cases, certain tissues and organs are damaged. When a virus infects the body, the virus can activate immune cells and promote their proliferation and differentiation. At the same time, immune cells can secrete cytokines and activate other immune cells to initiate immune responses, thereby resisting viral infection.

### Cellular metabolic reprogramming caused by viruses

Metabolic reprogramming refers to altering cellular metabolic pathways, including those involving glucose, lipids, and amino acids, to meet the bioenergetic and biosynthetic demands of cellular proliferation and growth [14]. Viruses exploit and reshape host metabolic processes upon infecting host cells to facilitate their replication and dissemination. To support these needs, viruses employ complex mechanisms to reprogram host cell metabolism [15]. These virus-induced metabolic changes can be categorized into seven hallmark features: (i) increased glycolysis and lactate production; (ii) upregulation of the PPP; (iii) enhanced glutaminolysis; (iv) alterations in mitochondrial function; (v) elevated lipid metabolism; (vi) modified amino acid metabolism; and (vii) changes in other biosynthetic and bioenergetic pathways. Different viruses induce distinct metabolic alterations to optimize conditions for their proliferation and spread (Table 1).

**Table 1.** Mechanisms of viral reprogramming of cell metabolism.

Virus Type	Virus Name	Viral protein	Targeted pathways	Metabolic reprogramming mechanisms	References
DNA viruses	KSHV	LANA	Aerobic glycolysis	Upregulation of PKM2 via the hypoxia-inducible factor-1α axis (HIF-1α)	[16,17]
		K1		K1 activates the PI3K/AKT/mTOR pathway	[18]
		vGPCR		vGPCR activates the TSC/mTOR/HIF axis to upregulate the expression of HIF-1α and HIF-2α	[19]
		K5		Regulates endocytosis of growth factor-binding receptor-associated tyrosine kinases	[20]
		v-miRNAs		Downregulation of EGLN2 and HSPA9 reduces mitochondrial biogenesis	[21]
		LANA	Glutamine breakdown	Upregulation of glutaminase (GLS) and glutamate via activation of c-Myc	[22,23]
		Kaposin A		Increased expression of metabotropic glutamate receptor 1	[24]
	HCMV	–	Aerobic glycolysis	Upregulation of glucose transporter (GLUT) 4	[25]
		–	Glutamine breakdown	Increased expression of GLS and glutamate dehydrogenase (GDH) enzymes	[26]
		–	Fat metabolism	Activation of sterol regulatory element binding protein 1 (SREBP1) induces adipocyte-like lipogenesis.	[27]
	EBV	LMP1	Aerobic glycolysis	Upregulation of GLUT1 expression and activation of the FGFR1 signaling pathway by activating the mTORC1/NF-κB signaling pathway	[28,29]
			Fat metabolism	Activation of SREBP1 induces adipocyte-like lipogenesis	[30]
		LMP2	Mitochondrial changes	Increased expression of the fission protein dynamin-related protein 1 (Drp1) by activating the Notch pathway	[31]
	HBV	HBP	Glycolysis	Initiation of glycolytic bypass via the FOXO3/miRNA-30 b-5p/MINPP1 axis	[32]
RNA viruses	HTLV-1	HBZ	Aerobic glycolysis	Upregulation of cellular transcription factor TAp73	[33]
	SARS-CoV-2	–	Aerobic glycolysis	By activating the mitochondrial ROS/HIF-1α axis	[34]
		–	Fat metabolism	Activate the signal transduction activity of PI3K/AKT/mTOR/S6K	[35,36]
		–	PPP	Increased expression of transketolase (TKT) and transaldolase 1 (TALDO1)	[37]
	HCV	–	Fat metabolism	Activation of SREBP	[38]

” >Kaposi’s sarcoma-associated herpesvirus (KSHV), human cytomegalovirus (HCMV), human T-lymphotropic virus type 1 (HTLV-1), reactive oxygen species (ROS), Latent Membrane Protein 1 (LMP1), and mechanistic target of rapamycin complex 1(mTORC1).

#### Virus regulation of cellular immune response

Upon viral invasion, host cells initiate a range of immune responses, including both innate and adaptive immunity [39]. Innate immunity, the body’s first line of defense, relies on the rapid recognition of pathogen-associated molecular patterns (PAMPs) by pattern recognition receptors (PRRs), including Toll-like receptors (TLRs), nucleotide-binding oligomerization domain-like receptors (NLRs), retinoic acid-inducible gene I (RIG-I)-like receptors (RLRs), and C-type lectin receptors (CLRs). Upon activation, these receptors trigger signaling cascades that lead to the release of inflammatory mediators and establish an antiviral state in surrounding cells [40,41]. Activated macrophages and dendritic cells secrete cytokines and chemokines that recruit additional immune cells to the infection site, forming a link between innate and adaptive immunity [42]. Adaptive immunity is a highly specific and long-lasting response mediated by T and B lymphocytes [43]. PRRs activation also stimulates the production of IFNs, particularly IFN-α and IFN-β, which bind to their receptors and activate the JAK-STAT pathway, thereby inducing the expression of interferon-stimulated genes (ISGs) [44]. These ISGs encode antiviral proteins that block multiple steps of viral replication. For example, IAV infection, an initial wave of IFN production initiates antiviral defenses, followed by a second wave of cytokines from innate immune cells that stimulate and regulate adaptive responses [45]. These processes ultimately promote viral clearance and help restore tissue homeostasis [46].

**Figure 2.**
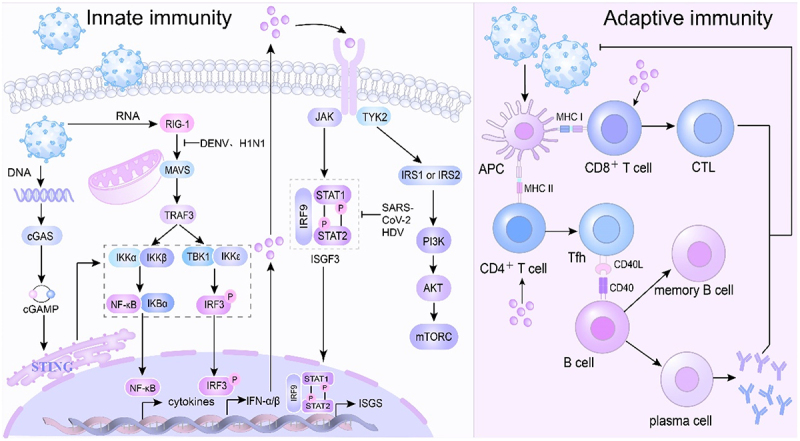
Virus-mediated immune response.

Despite these robust defenses, many viruses have evolved effective immune evasion strategies. In DENV infection, mutations within the envelope protein impair antibody binding, enabling the virus to evade the host immune system [47]. Beyond antigenic variation, numerous viruses produce immunosuppressive proteins that interfere with antigen presentation, T cell activation, and antibody production. DENV also disrupts IFN signaling by inhibiting the interaction between RIG-I and mitochondrial antiviral signaling protein (MAVS), leading to reduced type I IFN production and weakened antiviral responses [48,49]. Similarly, severe acute respiratory SARS-CoV-2 encodes several proteins, such as (non-structural protein 6) NSP6, NSP13, open reading frame 7a(ORF7a), and ORF7 b, that inhibit IFN production by blocking STAT1 and STAT2 phosphorylation [50,51]. The nucleoprotein (NP) of the H1N1 IAV (PR8 strain) interacts with MAVS and TOLLIP to induce mitochondrial autophagy, thereby disrupting MAVS-mediated signaling and promoting viral replication [52]. Hepatitis D virus (HDV) also suppresses IFN-α-mediated antiviral responses by inhibiting the phosphorylation of STAT1 and STAT2 [53]. Overall, these targeted strategies allow viruses to persist and replicate within the host while avoiding immune recognition and elimination (Figure 2).

During innate immune activation, host cells employ pathways such as cGAS-STING and RIG-I-MAVS to detect and respond to viral nucleic acids. The cGAS-STING pathway is a key sensor of aberrant cytoplasmic DNA. Upon viral entry, viral DNA activates cyclic cGAS, which catalyzes the synthesis of cyclic GMP-AMP (cGAMP) from ATP and GTP. Acting as a second messenger, cGAMP binds to the adaptor protein STING on the endoplasmic reticulum membrane, leading to the activation of transcription factors NF-κB and IRF3, which initiate the expression of antiviral genes. In contrast, the RIG-I-MAVS pathway is the principal sensor of cytoplasmic viral RNA. Recognition of viral RNA by retinoic RIG-I triggers signal transduction to MAVS, which subsequently activates downstream signaling complexes such as TBK1 and IKK. These complexes phosphorylate IRF3 and NF-κB, promoting the production of type I interferons (IFN-α/β). IFN-I then binds to the interferon-α/β receptor (IFNAR), initiating a signaling cascade via TYK2 and JAK kinases. This leads to the phosphorylation and dimerization of STAT proteins, which translocate to the nucleus and bind to specific DNA sequences to induce the expression of ISGs. In addition, TYK2 May also activate insulin receptor substrate (IRS), thereby engaging the PI3K-AKT-mTORC signaling pathway, which contributes to the regulation of immune responses and metabolism. Adaptive immunity is initiated when APCs process viral antigens and present them to T and B lymphocytes. CTLs are activated by viral peptides presented on major histocompatibility complex (MHC) class I molecules and subsequently eliminate infected cells. In parallel, CD4^+^ helper T cells (Th), activated via MHC class II molecules, support B cell activation. These helper cells facilitate B cell maturation into plasma cells and memory B cells via B cell receptor (BCR) engagement and co-stimulatory signals. Plasma cells then secrete virus-specific antibodies, which neutralize the pathogen and prevent reinfection. Despite the effectiveness of both innate and adaptive immune responses, viruses have developed diverse immune evasion strategies. For instance, DENV and H1N1 inhibit RIG-I-MAVS signaling, thereby attenuating IFN production. HDV and SARS-CoV-2 suppress downstream IFN signaling by blocking STAT1 and STAT2 phosphorylation, impairing ISG induction, and facilitating viral replication. These mechanisms collectively enable viruses to escape immune detection, persist in the host, and promote disease progression.

#### Effects of viral infection on cell organelles

Viral infections can impair the function of cellular organelles, such as mitochondria and the endoplasmic reticulum (ER), disrupting energy metabolism and protein synthesis and thereby altering the cellular microenvironment. Mitochondria are the primary sites of ATP production via oxidative phosphorylation, supporting essential cellular functions. Beyond energy metabolism, mitochondria also regulate apoptosis, signal transduction, and various metabolic pathways. Uniquely, mitochondria possess their own DNA and protein synthesis machinery, allowing for autonomous division and replication [54–56]. The ER, an extensive membrane network distributed throughout the cytoplasm, is essential for protein and lipid synthesis, intracellular transport, and calcium storage [57]. Viral infection often induces ER stress, a condition marked by the accumulation of misfolded or unfolded proteins that disrupt cellular homeostasis. In response, the ER activates signaling pathways that communicate with the nucleus, initiating protective mechanisms such as the unfolded protein response (UPR) or, if the stress is severe, apoptosis. These responses aim to restore ER function and limit viral replication. Furthermore, the HBV-encoded HBx protein activates the IRE1α-XBP1 signaling pathway, leading to ER stress, which contributes to the establishment of persistent viral infection.

DNA and RNA viruses employ distinct mechanisms to modulate organelle function and shape the cellular microenvironment in favor of viral replication. DNA viruses such as HBV induce mitochondrial perinuclear aggregation and promote mitochondrial fission through Drp1 phosphorylation. HBV also upregulates Parkin and PINK1, key regulators of mitophagy, thereby enhancing mitochondrial autophagy [58,59]. Furthermore, the HBV-encoded HBx protein activates the IRE1α-XBP1 signaling pathway, leading to endoplasmic reticulum (ER) stress, which contributes to the establishment of persistent viral infection [60]. Similarly, the p17 protein of ASFV promotes mitophagy by enhancing the interaction between SQSTM1 and TOMM70 [61]. HCMV increases Drp1 expression to drive mitochondrial fission and directly harnesses mitochondrial energy production to sustain its replication. RNA viruses also target mitochondrial and ER functions through various mechanisms [62]. RNA viruses also target mitochondria and the ER through various mechanisms. HCV for example, induces mitochondrial fission and mitophagy via Drp1 phosphorylation and triggers ER stress by activating the IRE1–XBP1–ERAD pathway through its E1 and E2 proteins [63,64]. The ORF9 b protein of SARS-CoV suppresses the innate immune response by promoting the degradation of the MAVS/TRAF3/TRAF6 signaling complex within mitochondria. Concurrently, the ORF8 protein impairs ER autophagy by binding to the autophagy receptor p62, leading to ER stress and damage [65,66]. DENV downregulates Drp1 through its NS4 B protein to inhibit innate immune responses [67]. Similarly, IAV impairs antiviral defenses by inhibiting both MAVS and the NLRP3 inflammasome [68,69]. Overall, these strategies demonstrate how viruses manipulate mitochondrial and ER functions to remodel the cellular microenvironment, enhancing viral replication and propagation (Table 2).

**Table 2.** Mechanisms of viral effects on cell organelles.

Virus Type	Virus Name	Viral protein	Effects on cell organelles	mechanism	Effects on cell physiology	References
DNA viruses	HBV	HBX	Enhances mitochondrial fission and mitophagy	Phosphorylation of Drp-1, upregulation of Parkin and PINK1	Inhibits apoptosis and innate immune responses, promoting persistent viral infection	[63]
			Inducing ER stress	Activation of the IRE1α-XBP1 pathway	Promotes persistent viral infection	[60]
	ASFV	p17	Induction of mitophagy	p17 promotes mitophagy by promoting the interaction between SQSTM1 and TOMM70	Suppressing innate immunity	[64]
	HCMV	—	Enhanced mitochondrial fission	Upregulation of Drp-1	Inhibit cell apoptosis	[62]
RNA viruses	HCV	—	Enhances mitochondrial fission and mitophagy	Phosphorylation of Drp-1	Inhibits apoptosis and innate immune responses, promoting persistent viral infection	[63]
		E1,E2	Inducing ER stress	Activation of the IRE1-XBP1-ERAD pathway	Promotes persistent viral infection	[64]
	SARS-CoV2	ORF-9b	Enhances mitochondrial fusion	ORF9b degrades MAVS/TRAF3/TFAF6 signalosome	Suppressing innate immunity	[65]
		ORF8	Inhibit ER autophagy and cause ER damage	ORF8 binds to p62	Promote viral replication	[66]
	MNoV	NS3	Permeabilizes mitochondrial membranes and induces mitochondrial dysfunction	The N-terminus of the NS3 protein binds to the mitochondrial membrane lipid cardiolipin.	Promotes persistent viral infection	[70]
	DENV	NS4B	Inhibits mitochondrial fission and induces mitochondrial elongation	Inhibition of Drp-1	Suppressing the innate immune response	[67]
	IAV	PB1-F2	Induction of mitochondrial fragmentation	Inhibits MAVS and NLRP3 inflammasome activation	Inhibition of antiviral responses via mitochondrial pathway	[68,69]

### Effects of the cellular microenvironment on viral infection

The influenza virus targets epithelial cells, endothelial cells, and alveolar macrophages, leading to the production of an initial wave of cytokines, including type I and type III IFNs, IL-1β, IL-18, TNF-α, IL-6, and IL-3. These cytokines act to restrict viral replication and dissemination while simultaneously initiating downstream immune responses [46]. When HBV enters the body, it can be recognized by T cells. CTLs can directly recognize and kill infected hepatocytes, while T cells can also secrete cytokines such as IFN and tumor necrosis factor (TNF) to control the virus [71,72]. Similarly, the porcine reproductive and respiratory syndrome virus (PRRSV) is captured by macrophages or dendritic cells and presented to T cells, initiating the host’s immune response [73]. DENV can stimulate mast cells to upregulate cytokines and chemotactic molecules, thereby promoting the activation of NK cells and NKT cells, which then exert antiviral effects [74].

The basement membrane is rich in LN and collagen. LN, first isolated in 1979 from Engelbreth-Holm-Swarm (EHS) mouse sarcoma, plays a critical role in cell adhesion, migration, and communication [75]. These functions are particularly relevant during the early stages of viral infection, as viral adhesion and migration are critical for host tissue invasion. For instance, LN5 acts as a transient receptor for human papillomavirus (HPV), facilitating its transfer between adjacent cells [76]. Similarly, the Lsa24 protein of Leptospira interrogans exhibits specific binding to LN [77]. Collagen, the most abundant protein in the ECM, comprises 28 members of the collagen superfamily and contributes to ECM structure by forming fibers, networks, and filaments [78,79]. It can modulate viral infection by influencing receptor accessibility, intracellular signaling pathways, and immune responses. For instance, in Caenorhabditis elegans, collagen forms a physical barrier in intestinal epithelial cells that prevents entry of the Orsay virus (OrV), thereby inhibiting infection. These examples underscore the ECM’s multifaceted role in regulating viral invasion and host-pathogen interactions [80].

Overall, Viruses can actively modify the microenvironment to facilitate their replication and dissemination by altering host cell metabolism, immune responses, and signaling pathways. In turn, the cellular microenvironment influences viral transmission and adaptability through immune-mediated responses and various physical and chemical factors.

## Viral infection and glucose metabolism

### Glucose metabolism

Glucose cannot enter cells via simple diffusion and instead requires specific transporter proteins, including GLUTs and sodium-dependent glucose transporters (SGLTs). These transporters are essential for maintaining glucose homeostasis across cellular membranes. Based on their structural and functional differences, GLUT proteins are classified into several subtypes, with GLUT1, GLUT2, GLUT3, and GLUT4 being the most extensively studied. Each subtype exhibits distinct expression patterns, affinities, and regulatory mechanisms. For instance, GLUT1 is widely expressed in various cell types, particularly in brain tissue and red blood cells, where it facilitates basal glucose uptake. GLUT2 is predominantly found in the liver, kidneys, and small intestine and plays a critical role in systemic glucose regulation and sensing. GLUT3, primarily expressed in the nervous system, has the highest affinity for glucose and ensures adequate glucose supply to neurons. GLUT4, which is mainly expressed in skeletal muscle and adipose tissue, is regulated by insulin and mediates insulin-stimulated glucose uptake. These transporters collectively ensure efficient glucose distribution to meet tissue-specific metabolic demands [81].

HK is one of the key enzymes initiating glucose metabolism by catalyzing the phosphorylation of glucose to glucose-6-phosphate (G-6-P), a critical metabolic intermediate that feeds into glycolysis, PPP, and glycogen synthesis. There are four HK isoforms, each exhibiting distinct expression patterns depending on the cell type. For instance, HK2 is predominantly expressed in bone and adipose tissues, whereas HK1 is the main isoform expressed in the normal brain. While HK1, HK2, and HK3 exhibit high affinity for glucose, HK4, also known as glucokinase, has a lower affinity and is active mainly at higher physiological glucose concentrations. Several viruses have been shown to manipulate HK activity to modulate host glucose metabolism in favor of viral replication [82]. Phosphofructokinase-1 (PFK-1) is another key regulatory enzyme in glycolysis, catalyzing the conversion of fructose-6-phosphate and ATP into fructose-1,6-bisphosphate and ADP [83]. Pyruvate kinase (PK) represents the third rate-limiting enzyme in glycolysis, facilitating the conversion of phosphoenolpyruvate to pyruvate. In mammalian cells, PK is expressed in four main isoforms: PKM1, PKM2, PKR, and PKL, each exhibiting distinct regulatory properties and tissue distributions. Lactate dehydrogenase (LDH) catalyzes the final step of anaerobic glycolysis, converting pyruvate to lactate. Accumulation of lactate can alter the pH of the cellular microenvironment, thereby influencing various physiological and pathological processes. Overall, viral manipulation of key glycolytic nodes such as HK, PFK-1, and LDH highlights the metabolic flexibility exploited by viruses to support replication and modulate the host microenvironment.

### Regulatory mechanisms of viral infection on glucose metabolism

**Figure 3.**
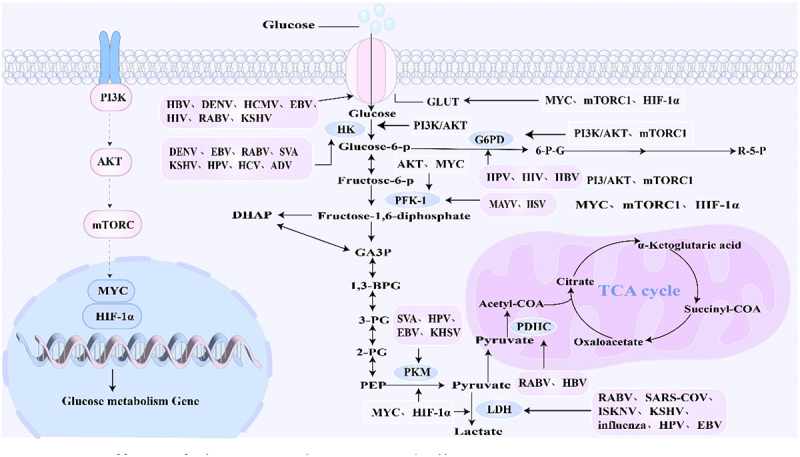
Effects of viruses on glucose metabolism.

Viruses require substantial energy to support their replication and proliferation. To meet this demand, viruses can reprogram host glucose metabolism by modulating glucose uptake, the activity of key metabolic enzymes, and associated signaling pathways (Figure 3).

The PI3K-AKT pathway is a key regulator of glucose metabolism. Various viruses, including HBV, DENV, HCMV, EBV, HIV, RABV, and KSHV, can modulate GLUT activity to influence glucose uptake and metabolism. GLUT activity can be affected by multiple factors such as MYC, mTORC1, and HIF-1α. In addition to GLUT, viruses can regulate the activity of other key metabolic enzymes. For instance, DENV, EBV, RABV, Senecavirus A (SVA), KSHV, HCV, and ADV alter the activity of HK, which is regulated by the PI3K-AKT axis. Similarly, MAYV and herpes simplex virus (HSV) can modulate the activity of phosphofructokinase 1 (PFK-1), which is regulated by AKT and MYC. Pyruvate kinase (PK), a key enzyme governed by MYC and HIF-1α, is targeted by SVA, HPV, EBV, and KSHV. HPV, HIV, and HBV can alter the activity of glucose-6-phosphate dehydrogenase (G6PD), which is controlled by PI3K/AKT and mTORC1. Additionally, RABV, SARS-CoV, ISKNV, KSHV, IVA, HPV, and EBV can influence the activity of lactate dehydrogenase (LDH), another enzyme regulated by MYC and HIF-1α. Finally, RABV and HBV can regulate the activity of the pyruvate dehydrogenase complex (PDHC), affecting the overall glucose metabolism.

#### Effects of cytoplasmic virus replication on glucose metabolism

The activities of HK, GAPDH, pyruvate dehydrogenase (PDH), isocitrate dehydrogenase (IDH), succinate dehydrogenase (SDH), and malate dehydrogenase (MDH) were significantly increased in the brains of mice infected with the DRV strain of rabies virus (RABV) [84]. HCV replication can downregulate the expression of GLUT1 and GLUT2, thereby reducing glucose uptake [85]. HCV NS5A can also enhance the activity of HK2 and promote glycolysis [86]. DENV can increase the expression of HK2 in human foreskin fibroblasts (HFF) cells, thereby altering glucose metabolism [87]. Mayaro virus (MAYV) infection manipulates glucose metabolism by activating 6-phosphofructo-1-kinase [88]. Similarly, SVA induced glycolysis in PK-15 cells by increasing HK2, 6-phosphofructokinase (PFKM), and PKM. DENV upregulates GLUT1 and HK2 to regulate glucose metabolism [89]. SARS-CoV-2 infects hepatocytes and regulates phosphoenolpyruvate carboxykinase (PEPCK) to regulate glucose metabolism. It can also affect GLUT expression by regulating the AMPK signaling pathway, thereby inducing host glucose metabolism reprogramming [34]. These observations collectively reveal that cytoplasmic viruses adopt diverse strategies to reprogram host glucose metabolism by selectively modulating both transporter expression and enzyme activity. Such metabolic reprogramming enhances viral replication efficiency and supports the energy and biosynthetic demands of infection.

#### Effects of nuclear replication of viruses on glucose metabolism

The LMP1 protein encoded by EBV activates the mTORC1/NF-κB signaling pathway, upregulating GLUT1 expression, which increases glucose uptake. It can also regulate glucose metabolism by activating mTORC2 through autocrine signaling via insulin-like growth factor 1 (IGF1) [28]. Additionally, LMP1 can activate the FGFR1 signaling pathway, further promoting aerobic glycolysis [29]. HCMV increases glucose uptake by upregulating GLUT4 expression, while enhancing PFK-1 activity to boost glycolysis, thereby altering glucose metabolism [90]. The E6/E7 proteins of HPV can regulate the IGF2BP2/m6A-MYC signaling pathway to manipulate glucose metabolism [91]. The viral interferon regulatory factor 1 (vIRF1) encoded by KSHV can upregulate and recruit the E3 ubiquitin ligase Kelch-like 3 (KLHL3) to degrade hnRNP Q1, thereby enhancing aerobic glycolysis [92]. Additionally, vFLIP and miRNA encoded by KSHV can suppress the expression of GLUT1 and GLUT3 by activating the NF-κB pathway, thus altering glucose metabolism. Furthermore, during latent infection of endothelial cells, KSHV activates the PI3K/AKT pathway, inducing aerobic glycolysis and lactate production [93]. HBV downregulates the expression of SIRT3 via the TLR2-NF-κB-PGC-1α axis, leading to a reduction in the expression of CS and PDHC in macrophages, thus inhibiting enzymes in the TCA cycle [94]. The SHBs protein encoded by HBV enhances hepatic gluconeogenesis by activating the cAMP/PKA/CREB signaling pathway [95]. Additionally, HBV can upregulate G6PD expression via Nrf2 activation mediated by HBx, promoting the PPP [96]. The E4ORF1 protein encoded by adenovirus activates MYC, thereby promoting glucose synthesis in host cells [97]. Overall, these viruses exploit host glucose metabolism by targeting GLUTs, glycolytic enzymes, and key metabolic regulators such as MYC, NF-κB, PI3K/AKT, and mTOR (Table 3).

**Table 3.** Mechanisms of viral effects on glucose metabolism.

Virus Type	Virus Name	Encoded viral protein	Virus replication site	Types of GLUT	Enzymes of sugar metabolism	mechanism	References
DNA viruses	KSHV	vIRF1	Nucleus	GLUT1,GLUT3	HK2	vIRF1 upregulates and recruits KLHL3 to degrade hnRNP Q1 via the ubiquitin-proteasome pathway, thereby destabilizing GDPD1 mRNA and leading to the induction of aerobic glycolysis.	[98]
		microRNA			PKM2	KSHV microRNA inhibits glycolysis by downregulating GLUT1 and GLUT3 via activation of the NF-κB pathway.	[92]
		flip			HK2	KSHV vFLIP inhibits glycolysis by downregulating GLUT1 and GLUT3 via activation of the NF-κB pathway.	[92]
	EBV	LMP1	Nucleus	GLUT1	HK2	Controlling aerobic glycolysis by upregulating Glut-1 transcription through the mTORC1/NF-κB axis	[28]
						PDHE1α nuclear translocation regulates glucose metabolism via the IGF1-mTORC2 axis.	[3]
	HBV	—	Nucleus	—	CS,PDHC	HBV regulates the TCA cycle by hyperacetylation of the citrate synthase/PDH complex through the TLR2-NF-κB-peroxisome proliferator-activated receptor γ coactivator 1α (PGC-1α) axis	[94]
		HBX		GLUT1	PEPCK,G6Pase	HBX activates iNOS to produce NO, which in turn activates JNK to upregulate hepatic gluconeogenesis genes.	[99]
		HBX		GLUT1	G6PD	HBX activates Nrf2 and thus activates G6PD transcription	[96]
		HBP		—	BPGM	HBp activates the miRNA-30 b-5p/MINPP1 axis to accelerate the conversion of glucose to lactate and 2,3-bisphosphoglycerate (2,3-BPG).	[32]
		SHBs		—	PECK	SHBs activate cAMP/PKA/CREB, thereby inducing the expression of gluconeogenic genes.	[95]
	HPV	E6/E7	Nucleus	GLUT1	HK2,PFKM,PDK1,LDHA	E6/E7 regulates MYC methylation by activating IGF2BP2	[91]
	ADV	E4ORF1	Nucleus	—	HK2,PFKM	E4ORF1 binds to MYC and enhances the binding of MYC to glycolytic target genes, leading to increased expression of specific glycolytic enzymes.	[97]
RNA viruses	RABV	—	Cytoplasm	GLUT1	HK,PDH,IDH,SDH,MDH LDHA	Upregulates GLUTI and enzymes involved in glucose metabolism to regulate glucose metabolism	[84]
	DENV	—	Cytoplasm	GLUT1	HK2	Upregulation of GLUT1 and HK2 to regulate glucose metabolism	[87]
	HIV	Tat	Cytoplasm	GLUT1	—	Tat protein enhances miR-150 expression, which then upregulates GLUT1 in HIV-infected cells.	[4]
		—			G6PD	Upregulates GLUT1 and G6PD to regulate glucose metabolism	[100]
	HCV	NS5A	Cytoplasm	—	HK2	NS5A protein enhances HK2 activity and alters glycolysis to promote HCV replication	[86]
	SVA	—	Cytoplasm	—	HK2,PFKM,PKM	SVA induces glycolysis to promote its replication	[89]

” >pyruvate kinase M2 (PKM2), citrate synthase (CS), glucose-6-phosphatase (G6Pase), bisphosphoglycerate mutase (BPGM)

### Effect of glucose metabolism on viral infection

#### Glucose metabolism provides energy for viral infection

In the context of COVID-19, hyperglycemia commonly seen in diabetic patients is associated with increased disease severity. This may be partly attributed to enhanced viral replication, as SARS-CoV-2 primarily relies on aerobic glycolysis to support its life cycle [34]. After H1N1 infection, glycolytic enzymes such as HK2 are upregulated, promoting glycolysis; inhibition of glycolysis results in reduced H1N1 replication [101]. Adenovirus utilizes its E4ORF1 protein to activate MYC, thereby enhancing the transcription of genes involved in glycolysis and the PPP, ultimately promoting viral propagation [97]. DENV is also highly dependent on glucose availability, and its replication is significantly reduced under glucose-deprived conditions [87]. In addition, host signaling molecules such as p38 MAPK have been implicated in promoting HCV replication [102]. Taken together, these findings highlight that glycolytic flux and glucose availability are critical determinants of viral replication efficiency, making host glucose metabolism a potential target for antiviral intervention.

#### Effects of intermediate metabolites produced by glucose metabolism on viral infection

**Figure 4.**
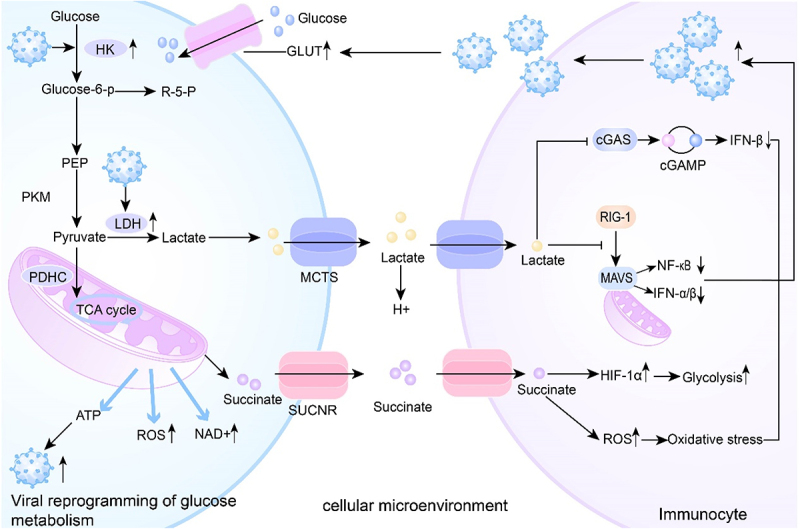
Interaction between the cellular microenvironment and glucose metabolism during viral infection.

Many viruses, including SVA, HBV, and PRRSV, can promote the conversion of pyruvate to lactate. The resulting accumulation of lactate binds to MAV, disrupting its localization to mitochondria and inhibiting the assembly of the RIG-I-MAVS signaling complex. This interference impairs MAVS aggregation, attenuates RLR signaling, and suppresses the production of IFNs [89,103–105]. Similarly, ASFV increases intracellular lactate levels, suppressing the cGAS – STING pathway and reducing IFN-β expression, ultimately enhancing viral replication [106]. HBx, a protein encoded by HBV, activates the NF-κB p65/HK2 signaling pathway to induce aerobic glycolysis. The lactate produced promotes the malignant proliferation of hepatocellular carcinomacells through the PI3K/AKT pathway [107]. EBV upregulates anaerobic glycolysis in B cells and increases LDH expression, thereby increasing lactate production. This lactate can inhibit the host antiviral response and enhance EBV replication and infection [108]. Beyond lactate-mediated effects, HBV can upregulate HK expression, which binds to voltage-dependent anion channels (VDAC) on mitochondria. This interaction facilitates the formation of a ternary complex with MAVS, impeding RIG-I-mediated signaling and IFN production [104,109]. IAV infection increases succinate in the host, which induces succinylation of IAV NP residue K87, preventing IAV from infecting cells [110]. Overall, these findings underscore that viruses not only reprogram glucose metabolism to meet their energetic and biosynthetic demands but also hijack metabolic intermediates like lactate and succinate to subvert host antiviral defenses (Figure 4).

Some viruses upregulate the expression of LDH, leading to increased lactate production. Lactate is then exported from the cell via lactate transporters, contributing to extracellular acidification through elevated hydrogen ion concentration. Simultaneously, lactate can reenter the cell and inhibit the RIG-I-MAVS signaling axis, a crucial component of the antiviral innate immune response. This inhibition suppresses the activation of NF-κB and the expression of interferons such as IFN-α and IFN-β, ultimately enhancing viral infection. Moreover, viral replication is directly fueled by host ATP, while elevated NAD^+^ levels can trigger inflammatory responses. The accumulation of ROS due to altered metabolism results in oxidative stress, further modifying the cellular environment. Additionally, increased succinate levels can activate its receptor, leading to the stabilization of HIF-1α, which enhances glycolytic activity and further supports viral proliferation. This reciprocal influence between metabolism and the cellular microenvironment underscores the importance of metabolic reprogramming in viral pathogenesis.

## Interaction between the cellular microenvironment and glucose metabolism during viral infection

Viral infection involves intricate interactions between the cellular microenvironment and glucose metabolism, which significantly influence the progression of the infection. Both the cellular microenvironment and glucose metabolism are crucial to the viral infection process. During infection, the cellular microenvironment undergoes alterations that can, in turn, affect glucose metabolism. Conversely, shifts in glucose metabolism can also modulate the cellular microenvironment. The subsequent discussion will explore these complex interactions through representative examples of viral infection mechanisms

HIV, a retrovirus responsible for acquired immunodeficiency syndrome (AIDS), primarily targets CD4^+^ T cells. HIV infection can promote the expression of GLUT1 and HK1, thereby inducing glycolysis. Glycolysis can activate HIF-1α signaling, which in turn facilitates HIV replication. HIV-1 dsDNA binding to CD4+T cells generates mitochondrial reactive oxygen species (mtROS). Furthermore, HIV-1 enhances the secretion of inflammatory cytokines via the HIF-1α signaling pathway, consequently altering the cellular microenvironment [111].

EBV, a gammaherpesvirus associated with several lymphoid and epithelial malignancies, plays a pivotal role in the pathogenesis of nasopharyngeal carcinoma (NPC). The EBV-encoded LMP1 promotes aerobic glycolysis by activating the PI3K/AKT pathway, which suppresses the GSK3β–FBW7 axis, leading to c-Myc stabilization. This stabilized c-Myc subsequently promotes HK2 gene expression and enhances glycolysis. The resulting glycolytic metabolites, such as lactic acid, contribute to the adhesion and growth of EBV-infected B lymphocytes by downregulating EBV-encoded microRNAs [108].

DENV, the causative agent of dengue fever and its severe forms, dengue hemorrhagic fever (DHF) and dengue shock syndrome (DSS), reprograms host metabolism upon infection. DENV activates the AKT/mTOR signaling pathway to facilitate its replication and enhances glycolysis to meet its biosynthetic needs. Simultaneously, infected immune cells ramp up glucose metabolism to support their effector functions, including cytokine production. In severe cases, this hyperactivation can trigger a cytokine storm, causing systemic inflammation and vascular leakage [112].

COVID-19, a highly infectious disease precipitated by SARS-CoV-2, poses a significant risk, particularly to individuals with diabetes, where dysregulated blood glucose levels are a major determinant of disease severity. SARS-CoV-2 infection triggers an increase in mROS production, which in turn stabilizes HIF-1α. The stabilization of HIF-1α promotes the upregulation of key glycolytic genes, including GLUT1, PKM2, and LDHA. This metabolic reprogramming contributes to a pro-inflammatory state in SARS-CoV-2-infected monocytes. Furthermore, succinate, an intermediate of glucose metabolism, enhances HIF-1α stability, thereby facilitating SARS-CoV-2 replication and infectivity [34].

In conclusion, the bidirectional regulation between glucose metabolism and the cellular microenvironment represents a crucial component of viral pathogenesis. Viruses hijack host metabolic pathways not only to fulfill their energetic and biosynthetic demands but also to manipulate immune responses by reshaping the microenvironment. While these mechanisms vary among viruses, understanding their shared and unique strategies can offer insights into disease progression and inform the development of novel antiviral therapies targeting host metabolism.

## Conclusion

Many viruses promote replication by altering host cell glucose metabolism and the cellular microenvironment. Research has shown that modulating glucose metabolism can effectively inhibit viral infections. For example, in the cases of SARS-CoV-2 and lymphocytic choriomeningitis virus (LCMV) infections, 2-DG can inhibit glycolysis, thereby reducing viral infection [113]. In HBV infections, metformin inhibits glycerol-3-phosphate dehydrogenase, thereby suppressing HBV replication [114]. Additionally, the cellular microenvironment influences viral replication. Hypoxic conditions are common features of many viral infections, promoting glycolysis and further enhancing viral replication through transcription factors like HIF-1α. For instance, D-mannose competitively inhibits glycolysis, reducing HIF-1α levels and thus diminishing virus-induced inflammation [115]. However, monotherapy has limitations, as 2-DG-induced inhibition of glycolysis may shift systemic metabolism toward lipid breakdown, potentially exacerbating conditions like ARDS [116]. This uncertainty in clinical application highlights the need for combined metabolic regulation and traditional antiviral therapies to improve treatment efficacy.

In recent decades, numerous studies have emphasized the key roles that the cellular microenvironment and glucose metabolism play in viral infections. These factors can trigger or suppress disease progression through various mechanisms. This paper provides an in-depth exploration of the regulatory mechanisms of cellular microenvironment and glucose metabolism during viral infections, aiming to enhance our understanding of viral pathogenesis. However, some complex issues in this field remain to be clarified. First, although some key mechanisms linking the cellular microenvironment and glucose metabolism during viral infections have been identified, our understanding of these mechanisms is still limited. For instance, further investigation is needed into how viral infections regulate glucose metabolism pathways and the generation of metabolic products, as well as how these metabolic changes influence the cellular microenvironment. Secondly, despite recognizing the importance of the cellular microenvironment and glucose metabolism in viral infections, effective therapeutic strategies remain lacking. Therefore, further exploration and development of novel antiviral strategies based on these interactions are needed to better address viral infections and related diseases, making significant contributions to improving human health.

## Data Availability

Data availability is not applicable to this article as no new data were created or analyzed in this study.
